# Development of RNAi method for screening candidate genes to control emerald ash borer, *Agrilus planipennis*

**DOI:** 10.1038/s41598-017-07605-x

**Published:** 2017-08-07

**Authors:** Thais B. Rodrigues, Lynne K. Rieske, Jian J. Duan, Kanakachari Mogilicherla, Subba R. Palli

**Affiliations:** 1 University of Kentucky, Department of Entomology, Lexington, 40546 USA; 2 USDA ARS Beneficial Insects Introduction Research Unit, Newark, Delaware USA

## Abstract

The ingestion of double-strand RNAs (dsRNA) targeting essential genes in an insect could cause mortality. Based on this principle, a new generation of insect control methods using RNA interference (RNAi) are being developed. In this work, we developed a bioassay for oral delivery of dsRNA to an invasive forest and urban tree pest, the emerald ash borer (EAB, *Agrilus planipennis*). EAB feeds and develops beneath the bark, killing trees rapidly. This behavior, coupled with the lack of a reliable artificial diet for rearing larvae and adults, make them difficult to study. We found that dsRNA is transported and processed to siRNAs by EAB larvae within 72 h after ingestion. Also, feeding neonate larvae with IAP (inhibitor of apoptosis) or COP (COPI coatomer, β subunit) dsRNA silenced their target genes and caused mortality. Both an increase in the concentration of dsRNA fed and sequential feeding of two different dsRNAs increased mortality. Here we provide evidence for successful RNAi in EAB, and demonstrate the development of a rapid and effective bioassay for oral delivery of dsRNA to screen additional genes.

## Introduction

The emerald ash borer (EAB), *Agrilus planipennis*, is an invasive forest pest that has caused the death of hundreds of millions of urban and forested ash trees (*Fraxinus* spp.) in North America^1^. EAB was accidentally introduced through solid wood packing material from Asia into the Detroit, Michigan (USA) area, where it was first discovered in 2002. Because of the prevalence of ash in urban areas throughout the region, the infestation established quickly and spread rapidly. Adult EAB is responsible for only minor feeding damage to ash foliage. However, larval feeding on cambial tissue beneath the bark disrupts water and nutrient transport to the canopy; tree death is rapid^2, 3^. All North American *Fraxinus* are susceptible, and EAB has more recently been reported on other Oleaceous hosts^4^. Therefore, development of efficient and target-specific products for EAB management is essential.

RNA interference technology is emerging as a next generation pest control method^5^ and as a new tool for integrated pest management (IPM). Double-stranded RNA (dsRNA) molecules activate the RNA interference (RNAi) pathway, a natural antiviral defense mechanism^6^ that processes long dsRNAs into small interference RNAs (siRNAs). These siRNAs are guided by RISC (RNA-induced silencing complex) to their complementary sequence in the messenger RNA (mRNA) which is cleaved and prevented from translation, resulting in a reduction in gene product; hence the gene silencing^7, 8^. Feeding dsRNA directly or through expression in transgenic plants to insects results in silencing of target genes and mortality^5^. However, RNAi efficiency varies among insects^9^. Critical species-specific factors must be considered in developing RNAi approaches, including the presence of RNAi machinery within the target pest, the length of the dsRNA fragment^10, 11^, the life stage of the target organism^12^, the target gene selected^13, 14^, and efficient delivery of the dsRNA^15^. The concentration of dsRNA^16^ and the combination of different dsRNAs used^17^ could also affect the efficiency of RNAi.

In EAB, in silico identification of RNAi pathway core component genes and the silencing of ScrB-2, a β-fructofuranosidase-encoding gene, after injection of dsRNA into adults has been reported^18^. However, for applications of RNAi for controlling EAB, the optimal target gene(s) and other delivery methods, including ingestion, need to be investigated. RNAi efficacy varies among insects. In hemipteran insects, the presence of dsRNases in their saliva has shown to degrade the exogenous dsRNAs, making oral delivery impractical^19, 20^. Interestingly, differences in RNAi susceptibility during different life stages of the same lepidopteran insect have also been observed^21^. Microinjection of dsRNA into larvae could be useful to screen candidate target genes and help to select genes that could be used in the RNAi-based control of EAB. However, the availability of large larvae is limiting mainly because: 1) the feeding behavior of endophagous insects such as EAB, which remain beneath the bark and develop inside the tree during all larval stages and 2) the lack of an artificial diet, which make efficient rearing of larvae in the laboratory outside host trees problematic. Also, microinjection of dsRNA into early stage larvae is difficult because they are very delicate and small. Thus, a feeding assay using neonate larvae would be an efficient and useful method to deliver dsRNA and rapidly screen potential target genes. Developing an oral delivery method using neonate larvae allows evaluation of the ability of EAB to process dsRNA and evaluation of RNAi effects throughout larval, pupal and adult stages.

The primary goals of our study were to evaluate the efficiency of RNAi machinery to process dsRNA into siRNAs and to develop an oral delivery method that would allow screening and selection of the most efficient candidate genes that cause larval mortality. We also evaluated sequential exposure to different dsRNAs and several dsRNA doses to improve RNAi efficiency in EAB.

## Results

### *In vivo* RNAi machinery

**Figure 1:**
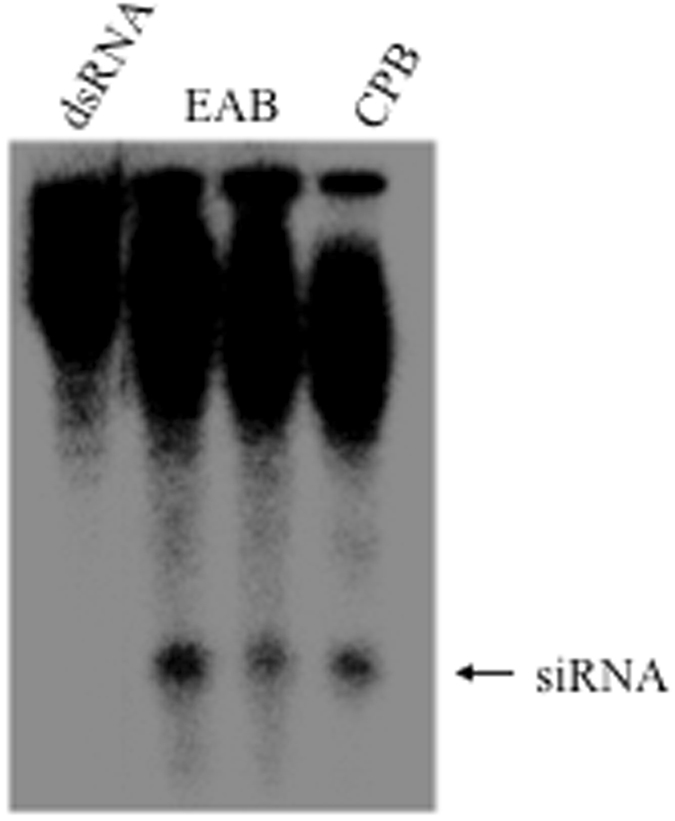
Transport and processing of dsRNA in EAB larvae. Total RNA isolated at 72 h after ^32^P labeled dsRNA injection was resolved on 8 M urea-20% polyacrylamide gel. The gel was dried and analyzed using a phosporImager. The left lane shows the intact dsRNA and the right lane shows the positive control CPB (RNA isolated from CPB larva fed on ^32^P labeled dsRNA). The middle two lanes show the RNA isolated from ^32^P labeled dsRNA injected EAB. Arrow point to siRNA band.

Three days following injection of ^32^P-labeled dsRNA into fourth instar EAB larvae, total RNA was extracted and resolved by Urea-acrylamide gel electrophoresis. A distinct band corresponding to the size of siRNA was observed in the RNA isolated from EAB larvae and showed the same intensity as observed for the positive control Colorado Potato Beetle (CPB, *Leptinotarsa decemlineata*), known to possess efficient RNAi machinery^22^ (Fig. 1), demonstrating that EAB contains machinery to transport dsRNA into cells and convert it to siRNA.

### Droplet feeding assay

**Figure 2:**
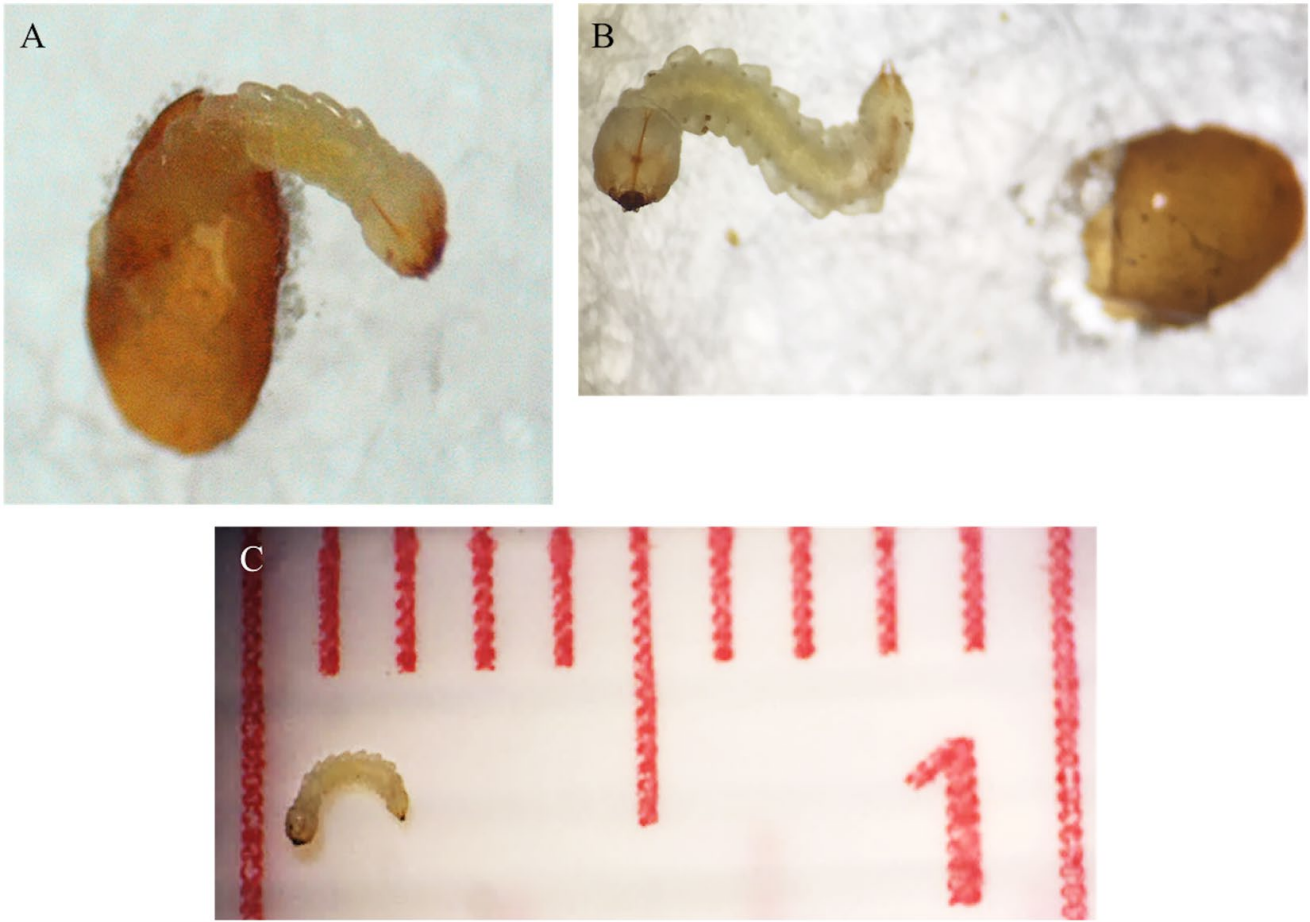
EAB neonate larvae. (**A**) EAB larva hatching from the egg; (**B**) Neonate larva size compared to egg; (**C**) neonate larva (~0.2 cm) on a scale of 1 cm. Olympus SZ61 Stereo Zoom Microscopy was used to amplify the EAB larvae and eggs and the pictures were taken by Olympus DP12 Microscope Digital Camera and iPhone 6S.

**Figure 3:**
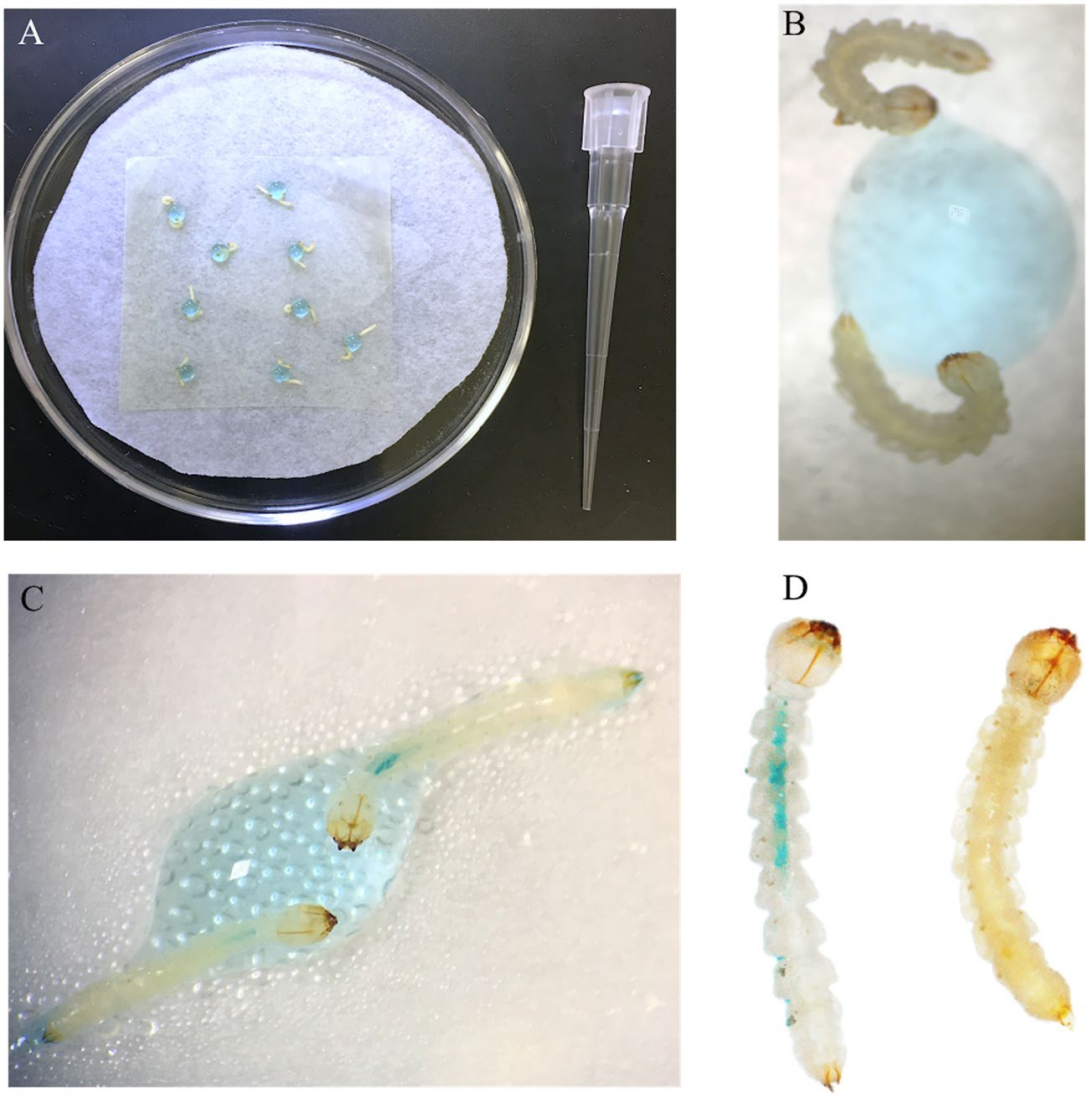
Droplet bioassay. (**A**) droplet feeding bioassay showing a pair of neonate EAB larvae feeding on a 2 uL drop of blue sucrose solution containing 6 ug/uL dsRNA and the 1μL pipette tip as scale; (**B**) Starting point of feeding assay and dsRNA exposure, 2 larvae per drop; (**C**) EAB larvae after feeding, showing intestinal tract with blue color; (**D**) Comparison between droplet fed larva (blue) and un-fed larva. Olympus SZ61 Stereo Zoom Microscopy was used to amplify the EAB larvae and the pictures were taken by Olympus DP12 Microscope Digital Camera and iPhone 6S.

To bypass the need for an artificial diet, a droplet feeding assay was developed to deliver dsRNA to EAB neonate larvae, which are fragile, 2 mm endophages (Fig. 2). The droplet assay uses dsRNAs in a sucrose solution with blue food dye. The sucrose provides an energy source to sustain the insects and the dye allows tracking ingestion of dsRNA in the larval intestinal tract (Fig. 3A–D). Because of their small size and endophagous habits, significant mortality (~48%) was observed in untreated larvae, necessitating the use of Sun-Shepard’s formula^23^ to adjust for mortality of control larvae. Larvae fed only the colored sucrose droplet were maintained for ten days, suggesting that the droplet assay would be most optimal on genes effective prior to neonate larval decline at day ten.

**Figure 4:**
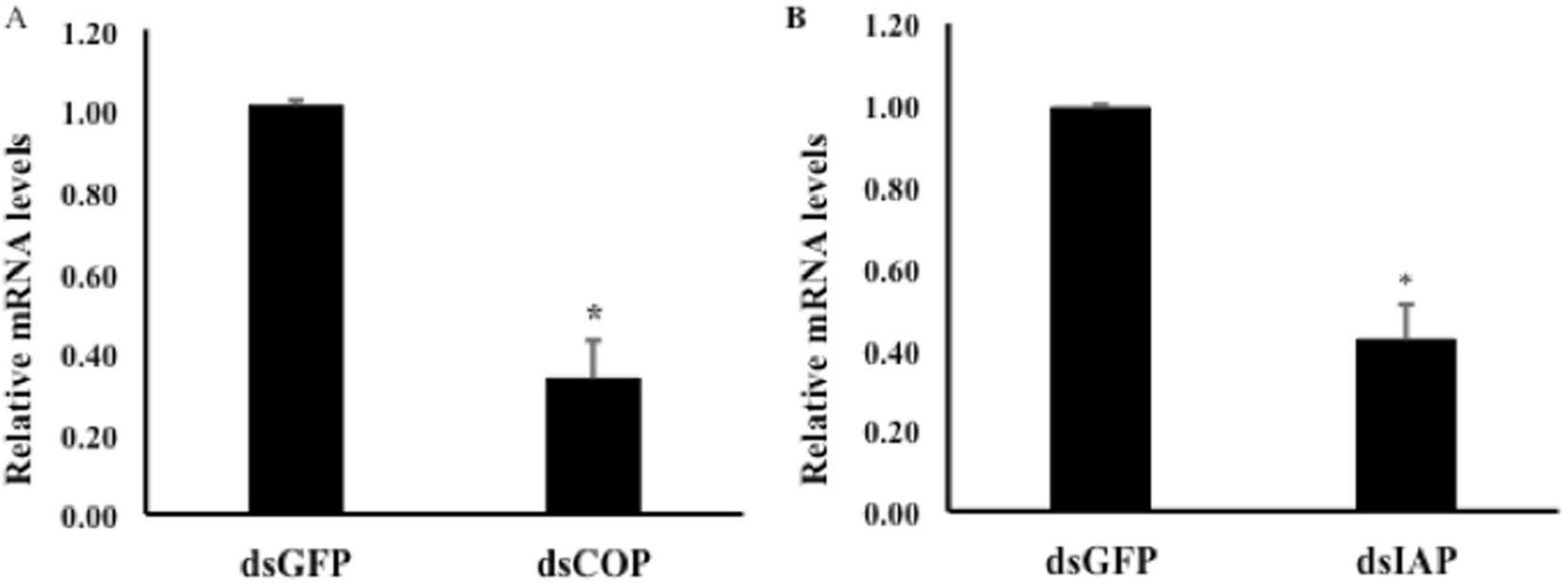
Relative expression of COP (**A**) and IAP (**B**) genes in neonate larvae exposed to 6 ug/uL dsRNA. The larvae were exposed at 6 ug/uL of dsCOP, dsIAP or dsGFP as a control. Total RNA was isolated on the 5^th^ day after initiation of feeding, and the mRNA levels of COP and IAP were determined using RT-qPCR. Mean + S.E (n = 3–4) are shown. The asterisk denotes treatments that are significantly different (t-test, one-tailed P-value: (**A**) P = 0.00112, (**B**) P = 0.001).

We tested two target genes COP (COPI coatomer, β subunit)^13, 14, 24^ and IAP (inhibitor of apoptosis 1)^25, 26^. Ingestion of dsIAP and dsCOP caused 57% and 67% knockdown respectively when compared to expression in control larvae (Fig. 4A,B). These results suggest that our droplet assay can deliver dsCOP and dsIAP to EAB larvae, and their cells can uptake these dsRNAs and suppress the expression of both COP and IAP genes.

**Figure 5:**
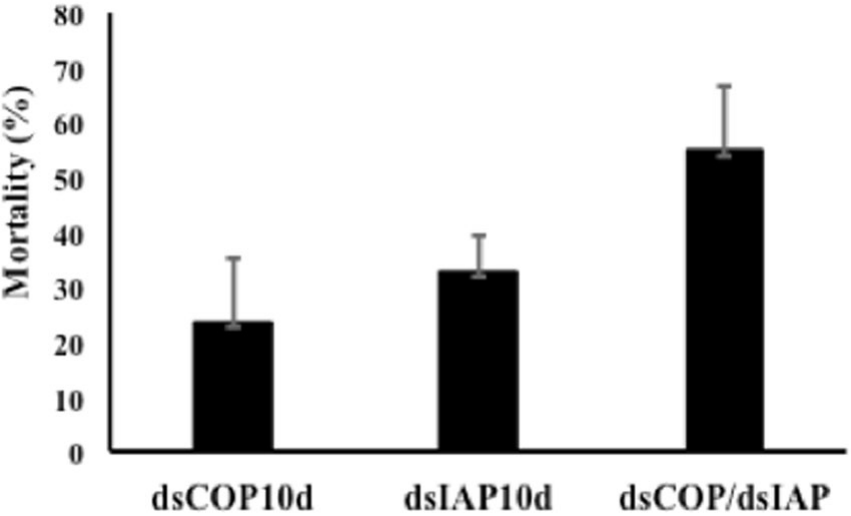
Larval EAB mortality after 10 days fed on single dsRNA and sequential dsRNAs. Neonates were exposed at 6 ug/uL dsRNAs for 4 consecutive days and then at blue-sucrose solution until day 10. dsCOP 10d:10 days on single dsCOPb; dsIAP 10d: 10 days on single dsIAP; dsCOPb/dsIAP: 2 days on dsCOPb plus 2 days on dsIAP, and the next six days fed on sucrose solution without dsRNA. The mortality was corrected using Sun-Shepard’s formula. Mean + S.E (n = 3–4) are shown. ANOVA, P = 0.141.

The analysis of the effect of knockdown of IAP and COP genes after ten days of exposure to respective dsRNA showed 33% and 24% mortality, respectively, following adjustment for control mortality (Fig. 5).

### Sequential exposure

**Figure 6:**
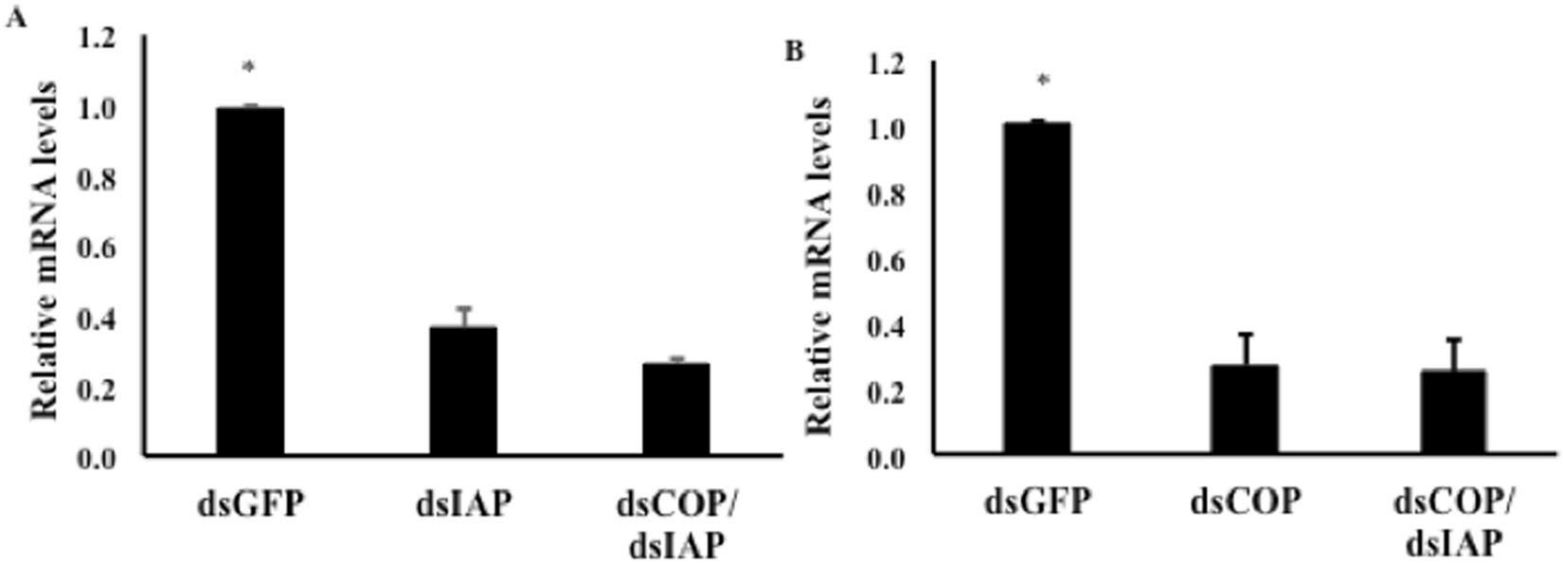
Relative expression of IAP (**A**) and COP (**B**) genes in EAB larvae on day 5, after 4 days feeding on 6 ug/uL of dsRNA. dsGFP: control; dsIAP and dsCOP: single exposure of dsRNA; dsCOP/dsIAP: sequential exposure of dsRNAs. For RT-qPCR, relative expression was measured and normalized to an endogenous control (TEF). Mean + S.E (n = 3–4) are shown. The asterisk above the bar indicates significantly different expression (ANOVA, Student-Newman-Keuls Method, P = < 0.001).

EAB larvae sequentially fed dsIAP and dsCOP for a total of four days had higher cumulative mortality after ten days (55%) compared to larvae fed only dsIAP (33%) or dsCOP (24%) (Fig. 5). Although neonate mortality rate was higher in larvae exposed sequentially to dsRNAs, no difference was detected in the expression of IAP or COP genes among treatments (Fig. 6A,B).

### dsRNA dose-response

**Figure 7:**
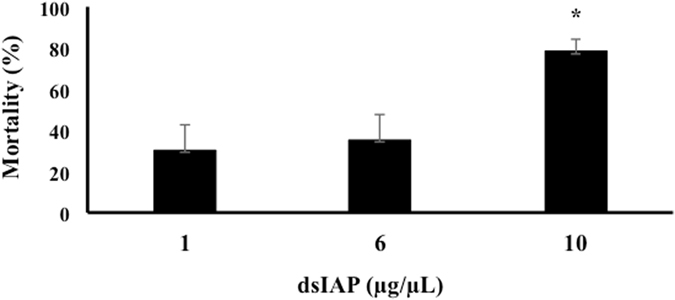
EAB mortality is evaluated after 10 days of feeding on dsRNA. Larvae were fed on three different concentrations of dsIAP for 4 days. 1:1 ug/uL; 6:6 ug/uL; 10:10 ug/uL of dsIAP. Values represent the means of the corrected mortality using Sun-Shepard’s formula. Mean + S.E (n = 3) are shown. The asterisk reflects significantly different mortality rate (ANOVA, Student-Newman-Keuls Method, P < 0.050).

Feeding on 10 μg/μL dsIAP caused 78% mortality, more than double the mortality caused by feeding on 1 μg/μL (30%) and 6 μg/μL (35%) of dsIAP (Fig. 7).

## Discussion

We demonstrate the existence of functional RNAi machinery in EAB larvae and develop a bioassay for rapid screening of target genes for use in RNAi-based control of this pest. A key component of the RNAi pathway is processing of long dsRNAs into small siRNAs (21–25 nucleotides). SiRNAs are the molecules that bind to the complementary mRNA, which silences the gene^7, 8^. In the present study, the processing of long dsRNA into siRNAs was investigated. Using microinjection we demonstrate that EAB larval cells are able to take up long dsRNAs and process them into siRNAs, similar to CPB where RNAi works well^27^ (Fig. 1). However, the delivery method of dsRNA by microinjection is not a practical approach for screening a large number of genes or for pest management. Therefore, a droplet-feeding assay was developed to orally deliver dsRNA to EAB neonate larvae (Fig. 3). Oral delivery of dsRNA provides several advantages^14^ in comparison to other delivery methods such as injection, transgenic plants, soaking, and transfection. Ingestion of dsRNA is applicable for high-throughput target gene screening, and is a less invasive and a more practical method for small insects and early instar larvae^14^. It is labor-saving, cost-effective, and easy. In our work, we confirmed the silencing of target genes (Fig. 4) and mortality after ingestion of dsRNA by EAB neonate larvae. However, some mortality was also observed in control larvae. We found that mortality in control insects increases with the length of the bioassay, possibly due to lack of essential nutrients. For example, at six days we observed 37.5% less mortality than at ten days (18% versus 48%). Therefore, this issue could be addressed by terminating bioassay on the sixth day.

We tested sequential exposure of two different dsRNAs (dsCOP followed by dsIAP) and we found a higher mortality compared to that caused by only dsIAP or dsCOP (Fig. 5), corroborating results from similar studies, which found that when *Tribolium castaneum* larvae are injected with combinations of two different dsRNAs, an increase in mortality is observed^17^. In that study two dsRNAs were injected simultaneously, which differed from our approach in which two dsRNAs were fed sequentially. It would be interesting to test for differences in efficacy of RNAi between sequential and simultaneous delivery of the same two dsRNAs in the same insect. Nevertheless, both experiments reached the same conclusion that treatment with two dsRNAs appears to have cumulative effects on insect survival. This conclusion is also supported by the analysis of COP and IAP gene expression in larvae fed on a single or two different dsRNAs. We found that the knockdown of IAP (Fig. 6A) and COP (Fig. 6B) genes in larvae fed on single dsRNA are the same as detected in larvae fed on two dsRNAs. Therefore, the increase in mortality may be explained by the cumulative effect of suppression of two essential genes simultaneously. The neonate larvae were also fed different concentrations of dsIAP and the higher concentration showed a higher mortality compared to mortality observed at the lower concentrations (Fig. 7). Previous work in *Bactrocera dorsalis* (Diptera: Tephritidae) showed that the concentration of dsRNA used influenced the level of gene suppression^28^. Although we analyzed the EAB phenotype effect instead of the gene expression level after feeding different concentrations of dsRNAs, a possible explanation for the increase in mortality observed may be due to higher suppression of the IAP gene at higher concentrations of dsRNA. Also, oral delivery of dsRNA has some limitations, such as natural barriers found in saliva and the gut environment in some insects^15, 29, 30^, including some coleopterans^31, 32^. Also, oral delivery makes it difficult to determine precise doses of dsRNA ingested by test insects^33^. These additional factors could help explain the need for high concentrations of dsRNA in the feeding bioassays in comparison to injection method^18^.

In conclusion, our findings confirm that *A. planipennis* possesses RNAi machinery to the knockdown target gene(s) after ingestion of dsRNA. We demonstrate that the droplet-feeding assay is an efficient method to orally deliver dsRNA to neonate larvae and quickly screen potential target genes by evaluating EAB neonate mortality. Our findings also provide a proof of concept that RNAi can be an option for EAB management. However, while our results raise hopes for the development of a new and highly specific method to control this devastating insect pest with greater efficiency and less off-target effects^34, 35^, they also showed the need for future research. A multitude of issues must be addressed before RNAi becomes feasible for EAB management. Essential steps for application of RNAi technology for EAB management include discovery of optimal target gene(s) that result in ~100% mortality of any stage, development of an applied system for widespread delivery of dsRNAs, such as foliar sprays, root absorption, trunk injections, or genetically modified trees expressing dsRNA targeting essential EAB genes. In addition, human health and environmental risk assessment to confirm the specificity of the product, its safety for off-target organisms and the fate of those molecules in the environment are also need to be performed.

## Methods

### Insects

Laboratory-reared *A. planipennis* eggs were placed in Petri dishes (150 × 15 mm) with moistened filter paper and maintained at 23 °C and 75% relative humidity in a growth chamber. Newly hatched neonates and larvae < 48 hr post-hatch were used in all bioassays.

### Evaluation of *in vivo* RNAi machinery

To evaluate the RNAi machinery in EAB larvae, we analyzed the processing of dsRNA into siRNAs by injecting ^32^P*-radiolabeled* dsRNA into last instar larvae. A 248 bp dsGFP was synthesized using the MEGAscript T7 kit and labeled with ^32^P UTP^36^. The labeled dsRNA was injected into larvae and 72 hr later total RNA was isolated and analyzed by Urea-polyacrylamide gel electrophoresis^36^. CPB larvae were also injected with labeled dsRNA and used as controls for RNAi efficiency^22^ and to identify processed siRNAs^27, 36^.

### Droplet-feeding bioassay

A droplet bioassay was developed to evaluate EAB susceptibility to RNAi and to screen for efficient target genes. Blue food coloring (Assorted Food Colors, Kroger Co., Cincinnati, OH USA) was added to confirm ingestion of test compounds by assessing the intestinal tract color change. A concentrated solution (4 μL of blue food coloring in 1 mL of 1% sucrose solution) was 2 × diluted before being offered to pairs of larvae as droplets on parafilm in a 50 × 9 mm petri dish containing moistened filter paper (Fig. 3A,B). Droplets consisted of 2 μL of blue sucrose solution with either dsRNAs or with nuclease-free water; droplets were replaced every other day until the end of each experiment.

EAB larvae were exposed to dsRNAs targeting COP (COPI coatomer, β subunit), and IAP (inhibitor of apoptosis), with GFP (green fluorescent protein) as a control. Each pair of larvae was exposed to 2 μL containing 6 μg/μL of dsRNA of each target gene for three days. On day four live neonates from each dsRNA treatment were transferred to new parafilm and fed on dsRNA for the next six days. EAB mortality (%) was recorded on the tenth day. Experiments were repeated 2–6 times under the same conditions, using 15 neonate larvae per replicate per treatment. The two genes that showed higher mortality compared to control were selected for further experiments to evaluate RNAi responses in EAB.

### Sequential exposure

Sequential exposure of EAB neonates to dsRNAs was used to analyze the difference in mortality of larvae fed on single dsRNA compared to a sequential exposure of two different dsRNAs. The experiment consists of three treatments and a control: 1) dsIAP alone for ten days; 2) dsCOP alone for ten days; 3) dsCOP for the first two days and dsIAP for next two days (four days of dsRNA exposure) and the next six days on sucrose solution without dsRNA; 4) dsGFP as the control. Larvae in all treatments were transferred to new parafilm and new dsRNA/sucrose solution every other day. Sun-Shepard’s formula^23^ was used to correct for mortality in controls (dsGFP). Four biological replicates were performed for the sequential exposure of dsRNAs (dsCOP plus dsIAP) and five for the single dsRNA (dsIAP 10d and dsCOP 10d). A one-way analysis of variance (ANOVA) was used for statistical analysis.

The knockdown of IAP and COP genes were analyzed for all the treatments. The larvae were fed on each treatment for four days. On day four, they were transferred to new parafilm and new sucrose solution without dsRNA and held for 24 hr. On the fifth day, live larvae were collected for RNA extraction and gene expression analysis as described below. Three and four biological replicates were performed, respectively, for the sequential exposure of dsRNAs (dsCOP plus dsIAP) and single dsRNA (dsIAP 10d and dsCOP 10d). A one-way analysis of variance (ANOVA) was used for statistical analysis, and Student-Newman-Keuls method was used to assess differences among treatments.

### dsIAP dose-response

Three concentrations of dsIAP (1, 6 and 10 μg/μL) were evaluated for neonate mortality, using a 10 μg/μL dsGFP as a control (the maximum concentration for the target gene). Larvae were fed on dsRNA for four days, followed by 6 days on blue sucrose solution without dsRNA. Mortality (%) was then calculated based on the initial number of larvae on day one. The experiment was performed three independent times. A one-way analysis of variance (ANOVA) was used with Student-Newman-Keuls to detect statistical differences.

### Target gene selection and synthesis of dsRNA

PCR templates for *in vitro* transcription of dsRNA were generated using gene-specific primers containing polymerase promoter sequence (TTAATACGACTCACTATAGGG) at the 5′end (Table 1). Candidate genes were selected based on the previous publications reporting mortality in insects exposed to those genes^37^. PCR conditions were 94 °C for 4 min, followed by 35 cycles of 94 °C for 30 s, 58 °C for 30 s and 72 °C for 45 s, finishing with an extension step at 72 °C for 10 min. The PCR template was purified using a PCR purification kit (Qiagen Inc., Valencia, CA USA). As a negative control, a fragment of GFP (green fluorescence protein) was amplified using T7 GFP primers. After PCR purification, dsRNA synthesis was performed using the MEGAscript RNAi Kit (Ambion Inc., Foster City, CA USA) following manufacturer’s instructions. Briefly, 200 ng of purified PCR product was used as template in a 20 μL *in vitro* transcription reaction. The reaction mix was incubated for 16 h at 37 °C, followed by 15 min of DNase treatment. The dsRNA was recovered adding 0.1 × volume of sodium acetate (3 M, pH 5.2) and 2.5x the volume of 100% ethanol and kept at −20 °C for at least 2 h following centrifugation at 4 °C for 30 min. The dsRNA pellet was then rinsed with 750 μL of 75% ethanol and centrifuged again at 4 °C for 15 min. The ethanol was removed and the dsRNA diluted in ultrapure distilled water. The quality of the dsRNA was checked by electrophoresis and quantified using a spectrophotometer (NanoDrop Technologies, Wilmington, DE USA).

**Table 1:** Primer sequence and amplicon size for the target genes IAP and COP. Bold letters represent the promoter sequence of T7 RNA Polymerase.

Primer Name	Primer Sequence (5′- 3′)	Amplicon (bp)	R^2^	Eff%
F-dsRNA-IAP	**TAATACGACTCACTATAGGG**CTCTAGAGATAGGAACGCACGGACAAT	272	—	—
R-dsRNA-IAP	**TAATACGACTCACTATAGGG**CCGCTCGAGACCGGTCTTCAAGCATCATC			
F-qRNA-IAP	CTTATCGCCGTACTGGGTGT	180	0.9	96
R-qRNA-IAP	GGAGGCTGCAACCATACACT			
F-dsRNA-COP	**TAATACGACTCACTATAGGG**AGGGCATGGGCAGTATTTA	247	—	—
R-dsRNA-COP	**TAATACGACTCACTATAGGG**AACTCGGTGTGGTCAGTT			
F- qPCR-COP	CAAAACGCCCGTTAGGATTA	151	0.9	102
R- qPCR-COP	CGGCACTCAGAACTTCAACA			

R^2^: Correlation Coefficients; Eff: Amplification efficiency.

### RT-qPCR

Total RNA was isolated from 6–10 EAB larvae pooled after feeding on dsRNA using the TRI Reagent RT (Molecular Research Center Inc., Cincinnati, OH, USA). The cDNA was synthesized using M-MLV Reverse Transcriptase (Life Technologies, Carlsbad, CA, USA) from 500 ng of RNA and used as a template for gene expression studies. The expression analyses of the target genes were conducted using SYBR Green PCR Master Mix. Briefly, the PCR mixture contained 1 μL of synthesized cDNA, 0.2 μL of each primer (10 mM), 5 μL of the SYBR green PCR master mix and 3.6 μL of ddH_2_O. The reactions were carried out in triplicate per template in a final volume of 10 μL. RT-qPCR reactions were performed on the StepOnePlus Real-Time PCR system (Life Technologies, Carlsbad, CA, USA) using the following cycling conditions: one cycle at 95 °C (20 s), followed by 40 cycles of denaturation at 95 °C (3 s), annealing and extension at 60 °C for 30 s. At the end of each RT-qPCR reaction, a melting curve was generated to confirm a single peak and rule out the possibility of primer-dimer and non-specific product formation. The TEF1A was used as reference gene^38^ and 2^−ΔΔCt^ method was used to calculate the relative expression level of the target gene in the samples as compared to controls^39^. A one-tailed t-test was used for statistical analysis to compare the mean of a single variable.
